# Supplementary material for: Reconstruction and visualization of large-scale volumetric models of neocortical circuits for physically-plausible in silico optical studies
Source: BMC Bioinformatics. 2017 Sep 13;18(Suppl 10):402. doi: 10.1186/s12859-017-1788-4 (PMC5606217; doi:10.1186/s12859-017-1788-4)
Supplement: Additional file 1 — High quality renderings of the generated 55 exemplar meshes and their morphologies. (PDF 4188 kb) [file 12859_2017_1788_MOESM1_ESM.pdf]

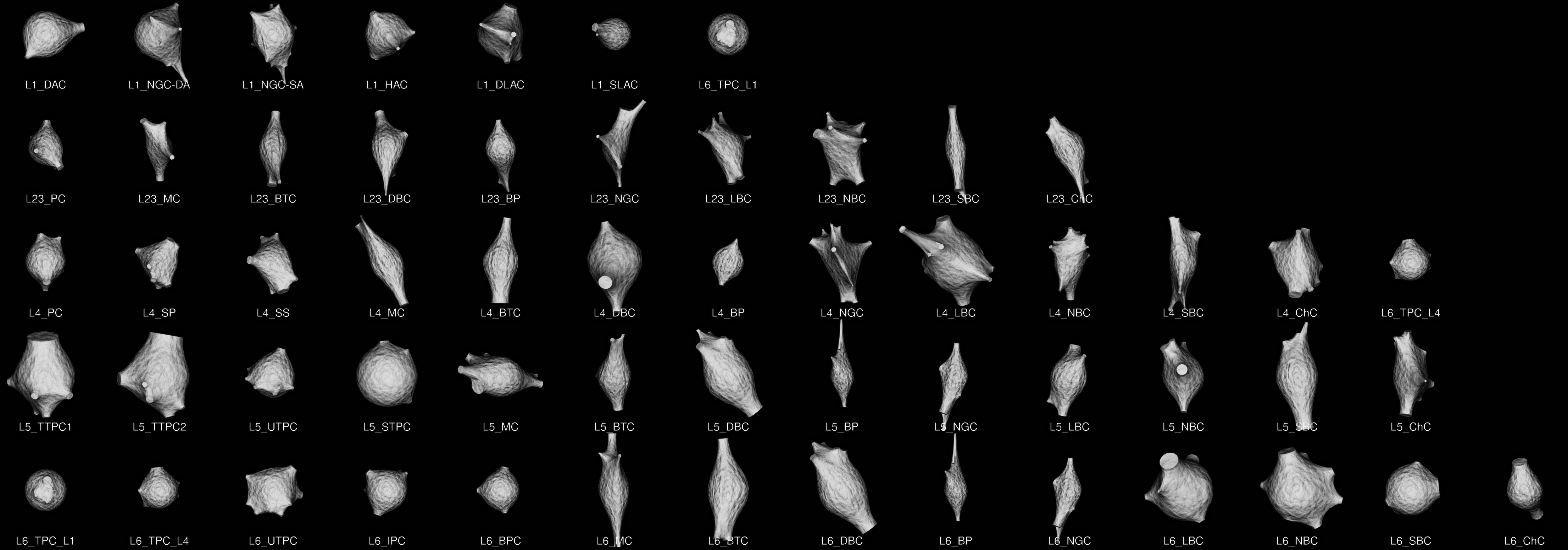
L1_DAC
L1_NGC-DA
L1_NGC-SA
L1_HAC
L1_DLAC
L1_SLAC
L6_TPC_L1
L23_PC
L23_MC
L23_BTC
L23_DBC
L23_BP
L23_NGC
L23_LBC
L23_NBC
L23_SBC
L23_ChC
L4_PC
L4_SP
L4_SS
L4_MC
L4_BTC
L4_DBC
L4_BP
L4_NGC
L4_LBC
L4_NBC
L4_SBC
L4_ChC
L6_TPC_L4
L5_TTPC1
L5_TTPC2
L5_UTPC
L5_STPC
L5_MC
L5_BTC
L5_DBC
L5_BP
L5_NGC
L5_LBC
L5_NBC
L5_SBC
L5_ChC
L6_TPC_L1
L6_TPC_L4
L6_UTPC
L6_IPC
L6_BPC
L6_MC
L6_BTC
L6_DBC
L6_BP
L6_NGC
L6_LBC
L6_NBC
L6_SBC
L6_ChC
